# Alkaptonuria

**Published:** 1899-05-20

**Authors:** 


### ALKAPTONURIA.

The very interesting paper read by Dr. Archibald Garrod at the last meeting of the Royal Medical and Chirurgical Society drew attention to a condition which, although of considerable rarity, should always be borne in mind when using Fehling's solution, from its tendency to simulate glycosuria by its reaction to that test. The urine of alkaptonuric individuals darkens after it is passed, rapidly acquiring a dark brown colour. This change becomes much more marked on the addition of an alkali, and it is attended by absorption of oyxgen. Fabrics moistened with such urine become deeply stained on exposure to the air. Such urine reduces Fehling's solution with the aid of heat, and ammoniacal silver nitrate solution in the cold, and its reducing effect may evidently lead to the condition being mistaken for one of glycosuria. The recognition of alkaptonuria is somewhat important, for it would appear from the cases which have been recorded that the condition is usually congenital and lifelong, and is apparently without injurious influence upon the health of the patient. Dr. Garrod gave a full account of the various theories which have been held from time to time in regard to the cause of alkaptonuria. The observations which oe recorded tended to support the view that the presence of homogentisinic acid was the essential feature of alkaptonuria.

